# Tenth International Medical Congress, Berlin, 1890

**Published:** 1890-03

**Authors:** 


ARTICLE III.

### TENTH INTERNATIONAL MEDICAL CONGRESS. BERLIN, 1890.

### REGULATIONS AND PROGRAMME.

I. The Tenth International Medical Congress will be opened in Berlin on Monday, August 4th, 1890, and will be closed on Saturday, August 9th.

II. The Congress shall consist of legally qualified medical men who have inscribed themselves as members, and have paid for their card of membership. Other men of science who interest themselves in the work of the Congress, may be admitted as extraordinary members.

Those who take part in the Congress shall pay a subscription of 20 marks (one pound st. or $5) on being enrolled as members. For this sum they shall receive a copy of the transactions, as soon as they appear. The enrollment shall take place at the beginning of the Congress. Gentlemen may, however, be enrolled as members by sending the amount of the subscription to the Treasurer Dr. M. Bartels, 75 Leipziger street, Berlin, SW., with their name, professional status and residence appended. (Please to enclose a visiting card.)

III. The object of the Congress is an exclusively scientific one.

IV. The work of the Congress will be discharged by eighteen different sections. The members shall declare upon enrollment to which section or sections they intend more particularly to attach themselves.

V. The Committee of Organization shall, at the opening sitting of the Congress, suggest the election of a definite committee (or bureau) which shall consist of a president, three vice-presidents, and a number—as yet undetermined—of honorary presidents and secretaries.

At the first meeting of each section a president and certain number of honorary presidents shall be elected; these latter shall conduct the business of the sections in turn with the president.

On account of the different languages employed, a suitable number of secretaries shall be chosen from among the foreign members. The duties of foreign secretaries shall be confined to the sittings of the Congress.

After the termination of the Congress the editing of the transactions shall be carried out by a committee, specially appointed for this purpose.

VI. The Congress will assemble daily, either for a general meeting or for the labors of the different sections.

The general meetings will be held between 11 and 2 o'clock. Three such meetings will take place.

The time for the sittings of the various sections will be fixed by the special committee of each section, it being understood, however, that no such sittings are to take place during the hours allotted to the general meetings.

Joint sittings of two or more sections may be held, provided that the bureau of the Congress can offer suitable rooms for such sittings.

VII. The general meetings shall be devoted to

*a*) Transactions connected with the work and general management of the Congress.

*b*) Speeches and communications of general interest.

VIII. Addresses in general sittings, as well as in any extraordinary meetings which may be determined upon can only be given by those who have been specially requested by the Committee of Organization.

Proposals relative to the future management of the Congress must be announced to the Committee of Organization before July 1st, 1890. The committee shall decide whether these proposals are suitable to be introduced for discussion.

IX. In the sittings of the sections, questions and problems will be discussed, which have been agreed upon by the special Committees of Organization. The communications of those appointed by the committee to report on a subject, shall form the basis of discussion. As far as time allows, other communications or proposals, proceeding from members and sanctioned by the Committee of Organization may also be introduced for discussion. The bureau of each section decides as to the acceptance of such offered communications, and as to the order in which they shall come before the meeting, always provided, that this point has not been already determined in the sitting itself by a decree of the section.

Scientific questions shall not be put to the vote.

X. Introductory addresses in the sections must as a rule not exceed *twenty minutes in length.* In the discussions no more than *ten minutes* are allowed to each speaker.

XI. All addresses and papers in the general and sectional meetings must be handed over to the Secretaries, in writing, before the end of the sitting. The Editorial Committee shall decide whether—and to what extent—these written contributions shall be included in the printed transactions of the Congress. The members who have taken part in the discussions, will be requested to hand over to the Secretaries, before the end of the day, in writing, the substance of their remarks.

XII. The official languages of all the sittings shall be German, English and French. The regulations, the programme and the agenda for the day will be printed in all three languages.

It will, however, be allowable to make use of other languages than the above for brief remarks, always provided that one of the members present is ready to translate the gist of such remarks into one of the official languages.

XIII. The acting president shall conduct the business of each meeting according to the parliamentary rules generally accepted in deliberative assemblies.

XIV. Medical Students, and other persons, ladies and gentlemen, who are not physicians but who take a special interest in the work of a particular sitting, may be invited by the President or be allowed to attend the sitting by special permission.

XV. Communications or enquiries regarding the business of separate sections, must be addressed to the managing members thereof. All other communications and enquiries must be directed to the General Secretary, Dr. Lassar, Berlin, NW., 19 Karlstrasse.

SPECIAL SECTIONS.—COMMITTEES OF ORGANIZATION.

(The names which appear in *italics* are those of the managing members.)

*1. Anatomy.*

Fleming, Kiel; Hasse, Breslau; *Hertwig*, Berlin, W., Maassenstr. 34; His, Leipzig; v. Kolliker, Wurzburg;

Kupffer, Munchen ; Merkel, Gottingen ; Schwalbe, Strassburg ; Wiedersheim, Frieburg.

2. *Physiology and Physiological Chemistry.*

Bernstein, Halle ; Biedermann, Jena ; *du Bois-Reymond*, Berlin, W., Neue Wilhelmstr. 15 ; Heidenbain, Breslau ; Hensen, Kiel ; Hufner, Tubingen ; Hoppe Seyler, Strassburg ; H. Munk, Berlin ; Voit, Munchen.

3. *General Pathology and Pathological Anatomy.*

Arnold, Heidelberg ; Bollinger, Munchen ; Grawitz, Greifswald ; Heller, Kiel ; Ponfick, Breslau ; v. Recklinghausen, Strassburg ; *Virchow*, Berlin, W., Schellingstr. 10 ; Weigert, Frankfurt, a. M.; Zenker, Erlangen.

4. *Pharmacology.*

Binz, Bonn ; Bohm, Leipzig ; Filehne, Breslau ; Jaffe, Konigsberg ; *Liebreich*, Berlin, NW., Dorotheen-Strasse 34a ; Marme, Gottingen ; Penzoldt, Erlangen ; Schmiedeberg, Strassburg ; Hugo Schulz, Greifswald.

5. *Internal Medicine.*

Biermer, Breslau ; Gerhardt, Berlin ; Leube, Wurzburg ; *Leyden*, Berlin, W., Theirgarten-Strasse 14 ; Lichtheim, Konigsberg ; Liebermeister, Tubingen ; Mosler, Greifswald ; Naunyn, Strassburg ; v. Ziemssen, Munchen.

6. *Diseases of Children.*

Baginsky, Berlin ; *Henoch*, Berlin, W., Bellevuestr. 8 ; Heubner, Leipzig ; Kohts, Strassburg ; Krabler, Greifswald ; Ranke, Munchen ; Ranke, Munchen ; Rehn, Frankfurt, a. M.; Soltmann, Breslau ; Steffen, Stettin.

7. *Surgery.*

Bardeleben, Berlin ; *v. Bergmann*, Berlin, NW., Alexander Ufer 1 ; Czerny, Heidelber ; Konig, Gottingen ; v. Lotzbeck, Munchen ; Schede, Hamburg ; C. Thiersch, Leipzig ; Trendelenburg, Bonn ; Wagner, Konigshutte.

8. *Obstetrics and Gynæcology.*

Fritsch, Breslau ; Gusserow, Berlin ; Hegar, Freiburg ; Hofmeyer, Wurzburg ; Kaltenbach, Halle ; Lohlein, Giessen; *Martin*, Berlin, NW., Moltkestr. 2; Olshausen, Berlin; Winckel, Munchen.

*9. Neurology and Psychiatry.*

Binswanger, Jena; Emminghaus, Freiburg; Erb, Heidelberg; Flechsig, Leipzig; Furstner, Heidelberg; Grashey, Munchen; Hitzig, Halle; Jolly, Strassburg; *Laehr*, Berlin-Zehlendorf.

*10. Ophtalmology.*

O. Becker, Heidelberg; Eversbusch, Erlangen; v. Hippel, Giessen; Hirschberg, Berlin; Leber, Gottingen; Michel, Wurzburg; Schmidt-Rimpler, Marburg; *Schweigger*, Berlin, NW., Roonstr. 6; v. Zehender, Rostock.

*11. Otology.*

Bezold, Munchen; Burkner, Gottingen; Kirchner, Wurzburg; Kuhn, Strassburg; Kessel, Jena; *Lucae*, Berlin, W., Lutzowplatz 9; Magnus, Konigsberg; Moos, Heidelberg; Trautmann, Berlin.

*12. Laryngology and Rhinology.*

Beschorner, Dresden; *B. Frankel*, Berlin, NW., Neustadtische Kirchstr. 12; Gottstein, Breslau; A. Hartmann, Berlin; Jurasz, Heidelberg; H. Krause, Berlin; Michael, Hamburg; Schech, Munchen; M. Schmidt, Frankfurt, a. M.

*13. Dermatology and Syphiligraphy.*

Caspary, Konigsberg; Doutrelepont, Bonn; Kobner, Berlin; *Lassar*, Berlin, NW., Carlstr. 19; Lesser, Leipzig; G. Lewin, Berlin; Neisser, Breslau; Unna, Hamburg; Wolff, Strassburg.

*14. Diseases of the Teeth.*

*Busch*, Berlin, NW., Alexander-Ufer 6; Calais, Hamburg; Hesse, Leipzig; Fricke, Kiel; Hollander, Halle; Miller, Berlin; Partsch, Breslau; Sauer, Berlin; Weil, Munchen.

*15. Hygiene.*

Flugge, Breslau; Gaffky, Giessen; Graf, Elberfeld; F. Hofmann, Leipzig; R. Koch, Berlin; Lehmann, Wurzburg;

*Pistor*, Berlin, W., v. d. Heydtstr. 13; Wolffhugel, Gottingen; Uffelmann, Rostock.

*16. Medical Geography and Climatology.*
(*History and Statistics.*)

Abel, Stettin; Brock, Berlin; Dettweiler, Falkenstein; Falkenstein, Lichterfelde; Finkelnburg, Bonn; Guttstadt, Berlin; *A. Hirsch*, Berlin, W., Potsdamer-Strasse 113; Lent, Koln; Wernich, Coslin.

*17. State Medicine.*

Falk, Berlin; Gunther, Dresden; v. Holder, Stuttgart; Knauff, Heidelberg; *Liman*, Berlin, SW., Koniggratzer-Strasse 46 a; Schonfeld, Berlin; Schwarz, Koln; Skrzeczka, Berlin; Ungar, Bonn.

*18. Military Hygieny.*

v. Coler, Berlin; v. Fichte, Suttgart; Grasnick, Berlin; Grossheim, Berlin; *Krocker*, Berlin, W., Magdeburger Platz 3; Mehlhausen, Berlin; Mohr, Munchen; Roth, Dresden; Wenzel, Berlin.

### CONDITIONS OF THE INTERNATIONAL MEDICO-SCIENTIFIC EXHIBITION.

I. The exhibition will be opened on the 2nd of August, at 11 o'clock a. m., and continue until the afternoon of the 11th, of August, 1890.

The exhibition will be held in the "Landes-Ausstellungs-Park," where also the meetings of the various sections of the Tenth International Medical Congress will take place,

It is intended to arrange dark-chambers and experimental-rooms, in which practical demonstrations for the benefit of the Congress, will be held, under the supervision of qualified persons.

The exhibits expected (as far as room allows,) will be as follows:

1. New or improved Scientific Instruments for Biological and Special Medical Purpose, including apparatus for

Photography and Spectral Analysis pertaining to medicine.

2. New Pharmacological Chemical Substances and Preparations.

3. New Pharmaceutical Substances and Preparations.

4. New Food Preparations.

5. New or improved Instruments for internal and external medicine, and allied specialties, including Electro-therapy.

6. Plans and Models (new) of Hospitals, Houses for reconvalescents, disinfection, and general Bathhouses.

7. New Appliances, such as pertain to nursing the sick, including methods of transportation, and baths for the sick.

8. Apparatus (new) for Hygienic Purposes.

9. Latest Medical Statistics and Compilations.

10. Scientific Preparations and Models pertaining to medicine.

11. Objects that pertain to Medical Instruction.

12. Literary (Scientific) Works.

II. The applications for space for exhibits must be made up to the 15th of May, 1890. Address Dr. O. Lassar, Secretary General, Bureau of the Tenth International Medical Congress, Berlin, N. W., Carlstrasse, No. 19.

Applications must be marked "Exhibition Affairs" and accompanied by a visiting card or card of the firm, on which the name and residence is plainly written or printed.

Applications must be made out in duplicate, and also, descriptive Lists of the articles to be exhibited, so that the exhibits can be eventually catalogued.

III. The heads of departments will decide whether and to what extent the articles for which application has been made may be exhibited.

In doubtful cases the Committee of Organization will decide.

IV. The rents for space must be paid to the Treasurer, Herrn Sanitatsrath, Dr. Bartels, Berlin, S. W., Leipzigerstrasse, No. 75, by postoffice money order as soon as the application has been accepted.

The prices for space is as follows:

For every square meter floor surface and part thereof ten marks.

For every square meter wall surface and part thereof six marks.

For floor surfaces which are adjacent to walls the exhibitor may make use of the wall surface to the height of two meters from the floor if he wishes to do so, without extra charge.

All objects that stand free with passages on each side, besides the floor surface allotted to the object (or objects) and virtually occupied by them, one half of the passage way on each side will also be charged for as floor surface occupied by said objects.

V. Tables will be furnished, but all cases and frames must be furnished by exhibitors, subject to the approval of the heads of departments.

For the use and furnishing of Motor Power, Electric Light, and special arrangements of every kind, the right to make special terms is reserved.

VI. All objects placed on exhibition will be insured free of charge, provided it is stated on the application, and the value of the object (or objects) given.

All substances or objects of an explosive or combustible nature will not be accepted for exhibition.

VII. Packing and unpacking will be done free of charge for foreign exhibitors, and in as careful a manner as possible, but no responsibility will be undertaken on the part of the Exhibition Committee. Native exhibitors must pay for packing and unpacking and look to the placing of their exhibits personally.

Messrs. Jacob & Valentine, Berlin, O., Holzmarktstrasse, No. 65, have undertaken the transportation.

VIII. Exhibits must be delivered in Berlin by the 20th of July.

On account of the Custom House formalities foreign exhibitors must obtain special formulas from the Exhibition Bureau before exhibits are sent.

IX. Exhibits cannot be removed before the close of the exhibition.

The special Committee on "Exhibition" consists of the following gentlemen: Commerzienrath, Paul Dorffel, H. Haensch; Director, Dr. J. F. Holtz; Director, L. Loewenherz; Regierungsrath, Dr. J. Petri, H. Windler and the Secretary General of the Committee of Organization. The names of the associate members of the Exhibition Committee as well as the names of the heads of departments will be made known shortly.

The following compose the Committee of Organization of the Tenth International Medical Congress: President, Dr. Rudolf Virchow; Vice-Presidents, Dr. E. von Bergmann, Dr. E. Keyden, Dr. W. Waldeyer; Secretary-General, Dr. O. Lassar.

INVITATION TO TAKE PART IN THE PROCEEDINGS OF THE SECTION FOR DISEASES OF THE TEETH.

In accordance with the resolution of the Ninth Congress held at Washington, the Tenth International Medical Congress will be held this year at Berlin, opening on the 4th of August, and continuing till the 9th of August, 1890. The delegates of the medical faculties and chief medical societies of the German Empire have elected us, the undersigned, as members of a Sectional Committee of Organization. In this capacity we have the honor cordially to invite your participation in the proceedings of our section. We hope to enjoy the satisfaction of welcoming large numbers of our colleagues to Berlin and also of seeing our section meetings numerously attended. We append the programme of our section, as far as hitherto fixed with the request that any further proposal, as well as offers of addresses, papers, or demonstrations, may be sent in with as little delay as possible.

(All communications or enquiries regarding the business of the section must be addressed to Professor Busch, Berlin, NW., Alexander-Ufer 6. Other communications and enquiries of a general character must be directed to the general secretary of the Congress, Dr. Lassar, Berlin, NW., Karlstr. 19.)

With the hope that the meetings of the section may prove interesting in themselves and useful to the advancement of science, we remain most resectfully,

The Committee of Organization for the Section of Diseases of the Teeth,

BUSCH, Berlin,
CALAIS, Hamburg,
HESSE, Leipzig,
FRICKE, Kiel,
HOLLANDER, Halle,
MILLER, Berlin,
PARTSCH, Breslau,
SAUER, Berlin,
WEIL, Munchen.

PRELIMINARY PROGRAMME OF THE DENTAL SECTION.

Monday, the 4th of August, 1790, after the close of the first general session, consitution of the dental section by the election of officers.

From Tuesday 5th, until Saturday 9th, inclusive, practical demonstrations will be given each day in the forenoon, between 9 and 12 o'clock, in the Dental Institute of the Royal University, Dorotheenstrasse 40.

These demonstrations will consist in extraction and narcosis, in filling and in artificial work. Gentlemen desiring to demonstrate either in extraction and narcosis or in artificial work are requested to apply to Professor Busch, (Alexander-Ufer 6.) Those who wish to demonstrate in filling are requested to apply to Professor Miller, (Vossstrasse 32.) For these demonstrations there are in the dental institute 15 chairs with good light, which number, in case of urgent necessity, might be increased to 19. The afternoons, between 2 and 5 o'clock will be devoted to theoretical lectures and discussions, in the hall of the Ressource zur Unterhaltung, Oranienburgerstr. 18.

The following five themes have been selected, the discussion of which will be opened by members appointed for the purpose.

1. Narcosis with bromide of ethyl in dental operations.

2. The cause, course and treatment of Pyorrhœa alveolaris.

3. The participation of micro-organisms in decay of the teeth.

4. Crown-and-bridgework.

5. The Bonwill method of articulation.

The members are also at liberty to present communications upon subjects with which they have particularly occupied themselves. Such communications along with a short resume of their contents should be announced to the chairman of the committee (Professor Busch, Alexander-Ufer 6.) On those days, on which general sessions are held, between 11 and 2 o'clock,) the practical demonstrations will be closed earlier and the theoretical lectures begun later.

EXTRACT FROM THE GENERAL REGULATIONS AND PROGRAMME.

IX. In the meetings of the sections, questions and problems will be discussed, which have been agreed upon by the special Committees of Organization. The communications of those appointed by the committee to report on subjects, shall form the basis of discussion. As far as time allows, other communications or proposals, proceeding from members and sanctioned by the Committee of Organization may also be introduced for discussion. The bureau of each section decides as to the acceptance of such offered communications, and as to the order in which they shall come before the meeting, always provided, that this point has not already been determined in the meeting itself by a decree of the section.

Scientific questions shall not be put to the vote.

X. Introductory addresses in the sections must as a rule not exceed twenty minutes. In the discussions no more than ten minutes are allowed to each speaker.

XI. All addresses and papers in the general and sectional meetings must be handed over to the secretaries, in writing, before the end of the meeting. The Editorial Committee shall decide whether—and to what extent—these written contributions shall be included in the printed transactions of the Congress. The members who have taken part in the discussions, will be requested to hand over to the secretaries, before the end of the day, in writing, the substance of their remarks.

XII. The official languages of all the meetings shall be German, English and French. The regulations, the programme and the agenda for the day will be printed in all three languages.

It will, however, be allowable to make use of other languages than the above for brief remarks, always provided that one of the members present is prepared to translate the gist of such remarks into one of the official languages.

Those who take part in the Congress shall pay a subscription of twenty marks (one pound sterling or five dollars) on being enrolled as members. For this sum they shall receive a copy of the transactions, as soon as they appear. The enrollment shall take place at the beginning of the Congress. Gentlemen may, however, be enrolled as members by sending the amount of the subscription to the Treasurer, Dr. M. Bartels, Berlin, SW., Leipzigerstr. 75, with their name, professional status and residence appended. (Please enclose a visiting card.)

### CONCERNING THE DENTAL SECTION OF THE TENTH INTERNATIONAL MEDICAL CONGRESS.

In response to a call of the organizing committee, Professors Virchow, von Bergmann and Waldeyer, fifty delegates from the various universities and medical societies of Germany met in Heidelberg on the 17th of September, 1888, to take steps in the organization of the Congress. At the meeting it was decided that the Congress should be held in Berlin, beginning August 4th, and closing August 10th, 1890.

An organizing committee consisting of Profs. Drs. Virchow, von Bergmann, Leyden and Waldeyer was elected and a general secretary, Dr. Lassar, appointed.

Eighteen sections, including dental Surgery, were organized, each with a special committee of nine members.

An international medico-scientific exhibition is to be connected with the Congress. Statutes and Programme were adopted which will be given in as far as they particularly concern the dental section.

ART. II. "The Congress consists of physicians, (approbirten ærzten), who have registered their names and obtained their membership cards. Other savants who are interested in the work of the Congress may be admitted as extraordinary members."

The delegates did not see fit to change this article so as to include dental surgeons, but decided that the article should be so interpreted as to admit dentists to membership. Since the meeting at Hiedelberg the question has been raised, whether dentists resident in Germany, but not possessing the German dental approbation degree, could be admitted to membership. Regarding this point the chairman of the committee of organization decided, that only those who possess the recognized degree of that country of which they are citizens, may be admitted to membership.

A German citizen holding only an American or Swiss degree, is therefore, not entitled to membership, no more is an American or English citizen not possessing the degree of his own country; on the other hand, foreign citizens practicing in Germany, are admitted without the German degree, provided, they have the degree of their own country.

Members pay a fee of twenty marks and receive a copy of the transactions.

ART. III. The object of the Congress is exclusively scientific.

ART. X. All lectures and communications in the general sittings or in those of the sections, must be handed in writing to the secretary before the close of the sitting. The editorial committee decides whether, or in what part such communications shall be included in the published transactions.

ART. XI. The official languages of all sittings are German, English and French. Very short remarks may be made in other languages, provided, some member is prepared to translate them into one of the official languages.

ART. XII. Lectures are, as a rule, to be limited to twenty minutes, discussional remarks to ten minutes.

ART. XIV. Students of medicine and other persons, gentlemen and ladies, who are not physicians, but are interested in the proceedings of any particular session, may be invited by the president of that session, or on application, receive permission to attend as auditors.

There are to be no vice-presidents associated with the congress, but each section is empowered to elect a limited number of honorary presidents and a secretary for each of the official languages.

The committee of the dental section is composed as follows: Busch, Berlin, Chairman; Calais, Hamburg; Hesse, Leipzig; Fricke, Kiel; Hollander, Halle; Miller, Berlin; Partsch, Breslau; Sauer, Berlin; Weil, Munich.

At a meeting of this committee, held on the 16th of October, 1889, it was decided that the hours from 9 to 12 A. M., should be devoted to practical demonstrations in the rooms of the dental institute, the demonstrations to consist of operations in filling, extraction and in mechanical dentistry, in short, operations in all branches of operative and mechanical dentistry.

Demonstrations in extraction and in artificial work are to be under the direction of Prof. Busch, those in filling under that of Prof. Miller. The theoretical exercises, &c., are to be held from 2 to 5. They will consist of the usual essays or lectures, and the accompanying discussions ; besides these, three subjects for general discussion are to be chosen, one to be introduced in the German language, (on bromide of ethyl by Prof. Dr. Hollander;) one in the English and one in the French language.

Those desiring to deliver lectures or read essays on particular subjects are requested to send in, along with their announcement, a very short resume of the contents of the same.

Correspondence in German language to be directed to Prof. Dr. Busch, chairman, Dorotheen street 40, Berlin ; in French language to Dr. Calais, Hohenbleichen 17, Hamburg ; in English language to Prof. Dr. Miller, Voss street 32, Berlin.

In America, Drs. Barrett and Taft ; in Great Britain, Mr. J. H. Mummery, M. R. C. S. and C., and Mr. W. Bowman Macleod, F. R. C. S., &c., have, on invitation of the committee, expressed their willingness to act in the capacity of honorary presidents.

A fuller report of the steps taken in the organization of the Congress up to the end of October, is given by Prof. Busch in the *Verhandlungen der Deutschen Odontologischen Gesellschaft*. Heft 2.

W. D. MILLER.

